# Bleeding Disorders in *Bothrops atrox* Envenomations in the Brazilian Amazon: Participation of Hemostatic Factors and the Impact of Tissue Factor

**DOI:** 10.3390/toxins12090554

**Published:** 2020-08-29

**Authors:** Sâmella S. Oliveira, Eliane C. Alves, Alessandra S. Santos, Elizandra F. Nascimento, João Pedro T. Pereira, Iran M. Silva, Jacqueline A. G. Sachett, Lybia Kássia S. Sarraff, Luciana Aparecida Freitas-de-Sousa, Mônica Colombini, Hedylamar O. Marques, Marcus V. G. Lacerda, Marco Aurélio Sartim, Ana Maria Moura-da-Silva, Luiz Carlos L. Ferreira, Ida S. Sano-Martins, Wuelton M. Monteiro

**Affiliations:** 1 Carlos Borborema Clinical Research Institute, Dr. Heitor Vieira Dourado Tropical Medicine Foundation, Manaus 69040-000, Brazil; oliveira.samella@gmail.com (S.S.O.); anealves.enf@gmail.com (E.C.A.); alessandrasantos910@gmail.com (A.S.S.); elyfreitas46@gmail.com (E.F.N.); pedro02.tavares@gmail.com (J.P.T.P.); iramend3@gmail.com (I.M.S.); jac.sachett@gmail.com (J.A.G.S.); lybsarraff@gmail.com (L.K.S.S.); marcuslacerda.br@gmail.com (M.V.G.L.); ana.moura@butantan.gov.br (A.M.M.-d.-S.); ferreira.luiz@gmail.com (L.C.L.F.); 2 School of Health Sciences, Amazonas State University, Manaus 69065-001, Brazil; 3 Immunopathology Laboratory, Butantan Institute, São Paulo 05503-900, Brazil; luciana.sousa@butantan.gov.br (L.A.F.-d.-S.); monica.colombini@butantan.gov.br (M.C.); 4 Hemostasis Laboratory, Amazonas State Hematology and Haemotherapy Hospital Foundation, Manaus 69050-001, Brazil; hedyomarques@gmail.com; 5 Institute of Biological Sciences, Federal University of Amazonas, Manaus 69070-000, Brazil; marcosartim@hotmail.com; 6 Pathophysiology Laboratory, Butantan Institute, São Paulo 05503-900, Brazil

**Keywords:** *Bothrops atrox* envenomation, coagulation factors, systemic bleeding, thrombocytopenia, tissue factor

## Abstract

Bleeding is a common hemostatic disorder that occurs in *Bothrops* envenomations. We evaluated the changes in coagulation, fibrinolysis components, and platelets in *Bothrops atrox* envenomations with bleeding. This is an observational study with *B. atrox* snakebite patients (*n* = 100) treated in Manaus, Brazilian Amazon. Bleeding was recorded on admission and during hospitalization. We found that the platelet count in our patients presented a weak correlation to tissue factor, factor II, and plasminogen. Tissue factor presented weak correlation to factor V, II, D-dimer, plasminogen, alpha 2-antiplasmin, and moderate correlation to fibrinogen and fibrin/fibrinogen degradation product (FDP). Patients with systemic bleeding (*n* = 20) presented low levels of factor V, II, fibrinogen, plasminogen, and alpha 2-antiplasmin, and high levels of tissue factor and FDP compared to those without bleeding. Patients with only local bleeding (*n* = 41) and without bleeding showed similar levels of hemostatic factors. Thrombocytopenia was observed mainly in patients with systemic bleeding and increased levels of serum venom. No association was found between venom levels and systemic bleeding, or between venom levels and clinical severity of envenomation. This is the first report that shows the participation of the extrinsic coagulation pathway in the consumption coagulopathy of *B. atrox* envenomations with systemic bleeding due to tissue factor release.

## 1. Introduction

Systemic bleeding following *Bothrops* envenomations is a common hemostatic disorder that has been observed in fatal envenomation cases in the Brazilian Amazon [1,2]. The lancehead (*Bothrops atrox*) is the most common venomous snake in the Amazon region [3] and hemostatically active toxins present in its venom are capable of converting fibrinogen into fibrin and activating coagulation factors, as well as interfering in platelet function, fibrinolysis, and endothelial maintenance [4,5,6,7,8,9,10,11,12,13,14,15]. The outcome is the installation of a consumptive coagulopathy, which is characterized by the depletion of hemostatic components. This depletion leads to unclottable blood and disruption of capillary vessels, which may be followed by bleeding [16,17]. 

A previous study in the Pará State, eastern Brazilian Amazon, has shown that *Bothrops* envenomations cause decreased levels of fibrinogen and alpha 2-antiplasmin, elevated fibrin/fibrinogen degradation product (FDP), and D-dimer concentrations [18]. Similar results were also reported in a study involving *Bothrops* snakebite patients in the Amazonas State, western Brazilian Amazon, in which some coagulation components recovered within 48 h after starting antivenom therapy [19]. Despite the clinical similarities of *Bothrops* envenomations in Brazil, thrombocytopenia is an infrequent event in *Bothrops* envenomation that occurs in the Amazon (caused mainly by *B. atrox*) compared to those in the southern region (caused by *B. jararaca*) [18,19,20]. Nevertheless, it has been shown that unclottable blood and thrombocytopenia are factors that are independently associated with the risk of systemic bleeding in *Bothrops* envenomations in the Brazilian Amazon [16]. 

Differences have been observed in the coagulant activity of venom from *B. atrox* snakes from distinct locations [21,22], habitats [23], and at different ontogenetic stages [24,25], as well as when compared to the venom of other *Bothrops* species [26]. There is little information regarding how clotting factors and other components are affected in *Bothrops* snakebite patients in the Amazon who evolve to systemic or local bleeding. Therefore, the aim of the present study was to evaluate the changes in coagulation factor levels, fibrinolysis components, and platelet count associated with bleeding observed in *B. atrox* snakebite patients in the western Brazilian Amazon. Our data show that *B. atrox* envenomation with systemic bleeding presents consumption of coagulation factors, such as fibrinogen, factors II and V, and fibrinolytic components, as well as the participation of tissue factor. 

## 2. Results

### 2.1. Patient Characterization

A total of 61 of the 100 patients included in our study presented showed bleeding on admission (before antivenom therapy) or during hospitalization (after antivenom therapy). Background information and clinical features of patients on admission based on the presence of bleeding are shown in Table 1. Twenty patients showed systemic bleeding and, of these, fourteen (70.0%) presented concomitant local bleeding. Nineteen (95.0%) of the patients that had systemic bleeding also had unclottable blood, and five (25.0%) were thrombocytopenic, including one patient in this group who had a platelet count of below 100 × 10^3^/µL. In four out of twenty (20.0%) patients with systemic bleeding, both unclottable blood and thrombocytopenia were observed. In patients with only local bleeding (41/61, 67.2%), twenty-one (51.2%) presented unclottable blood, and two (4.9%) thrombocytopenia, who also had unclottable blood. In addition, thirty-nine patients showed no bleeding after the *B. atrox* envenomation; of these, fourteen (35.9%) had unclottable blood and three (7.7%) presented thrombocytopenia. In two out of thirty-nine (5.1%) patients without bleeding both unclottable blood and thrombocytopenia were observed.

### 2.2. Coagulation Factors Levels and Platelets Count

**Figure 1:**
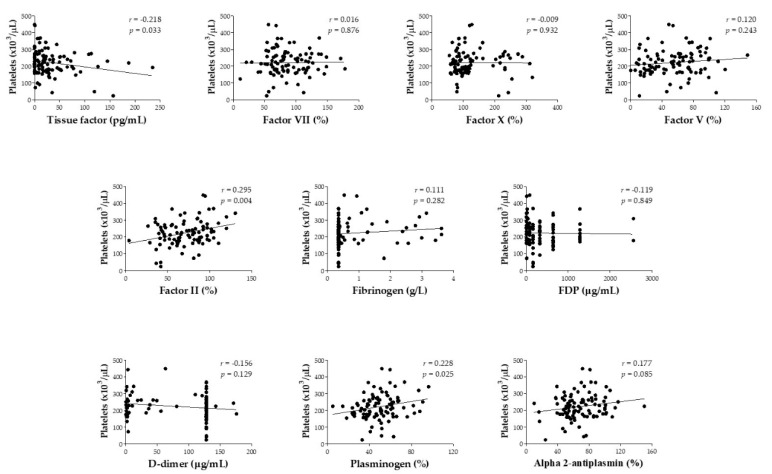
Pearson’s correlation (*r*) between platelet count and coagulation factor levels and fibrinolysis components obtained on admission (before antivenom therapy) for *Bothrops atrox* snakebite patients in the Brazilian Amazon.

Figure 1 shows correlation between platelet count and coagulation factor levels and fibrinolysis components on admission (before antivenom therapy) for *B. atrox* snakebite patients in the Brazilian Amazon. Platelet count presented weak correlation to tissue factor concentration (*r* = −0.218; *p* = 0.033), factor II (*r* = 0.295; *p* = 0.004), and plasminogen activity (*r* = 0.228; *p* = 0.025).

**Figure 2:**
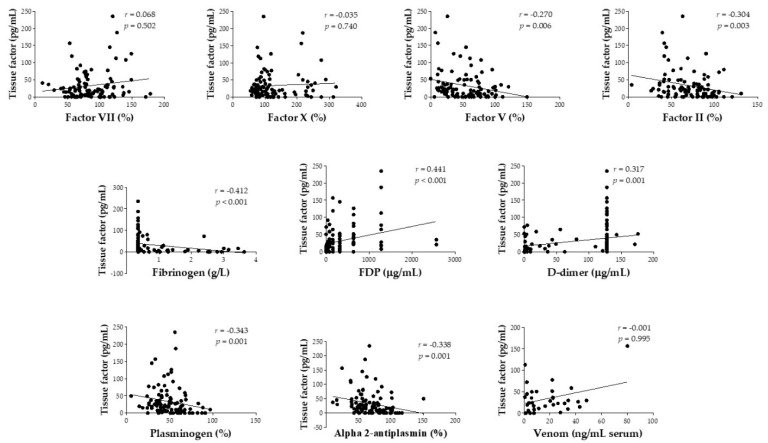
Spearman’s correlation (*r*) between tissue factor and coagulation factor levels/ fibrinolysis components and serum venom obtained on admission (before antivenom therapy) for *Bothrops atrox* snakebite patients in the Brazilian Amazon.

The correlation between tissue factor and coagulation factor levels/fibrinolysis components and serum venom on admission (before antivenom therapy) for *Bothrops atrox* envenomation was also evaluated in our study. Tissue factor presented a weak correlation to factor V (*r* = −0.270; *p* = 0.006), factor II (*r* = −0.304; *p* = 0.003), D-dimer (*r* = 0.317; *p* = 0.001), plasminogen (*r* = −0.343; *p* = 0.001), alpha 2-antiplasmin (*r* = −0.338; *p* = 0.001), and moderate correlation to fibrinogen (*r* = −0.412; *p* < 0.001) and FDP (*r* = 0.441; *p* < 0.001) (Figure 2). Tissue factor levels were also significantly increased in patients with moderate/severe edema (median = 25.8 pg/mL (IQR: 14.50–52.20)) when compared to those with mild edema (median = 8.3 pg/mL (IQR: 0.00–29.20)) (*p* = 0.003).

### 2.3. Bleeding vs. Coagulation Factors and Platelets

**Figure 3:**
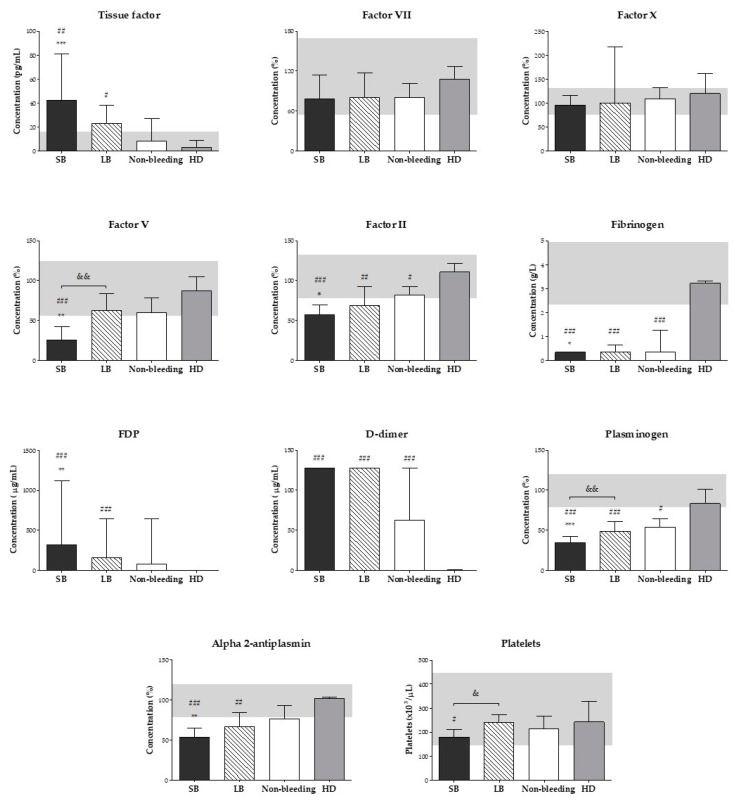
Plasma concentration of coagulation factors and fibrinolysis components, and platelet count on admission (before antivenom therapy) in patients with systemic bleeding (SB) (*n* = 20), only local bleeding (LB) (*n* = 41) and non-bleeding patients (*n* = 39) after *Bothrops atrox* envenomation in the Brazilian Amazon. Results are expressed as medians and interquartile ranges. *** *p* < 0.001, ** *p* < 0.01 and * *p* < 0.05, significant between patients with bleeding (SB or LB) and non-bleeding patients. ^&&^ *p* < 0.01 and ^&^ *p* < 0.05, significant between patients with systemic bleeding and only local bleeding. ^###^ *p* < 0.001, ^##^ *p* < 0.01 and ^#^ *p* < 0.05, significant between *B. atrox* snakebite patients (SB, LB, or non-bleeding patients) and healthy donors (HD; *n* = 14), who were accompanying patients during hospitalization (control group). FDP: fibrin/fibrinogen degradation product. The shaded area is the normal range for each parameter. Normal range: FDP < 5 µg/mL; D-dimer ≤ 0.5 µg/mL.

Figure 3 shows the median levels of coagulation factors, fibrinolysis components, and platelet count in *B. atrox* snakebite patients with either systemic bleeding, only local bleeding, or without bleeding and data for the healthy donor (HD) control group. Lower levels of factor II, fibrinogen, plasminogen, and alpha 2-antiplasmin, as well as higher levels of tissue factor, FDP, and D-dimer were observed in *B. atrox* snakebite patients with bleeding (systemic bleeding and only local bleeding) when compared to the HD control group. Levels of factor V were significantly decreased only in the patients with systemic bleeding. Patients with systemic bleeding showed lower levels of factor V, factor II, fibrinogen, plasminogen, and alpha 2-antiplasmin, as well as higher levels of tissue factor and FDP when compared to those without bleeding. There was no statistically significant difference in median levels of coagulation factors and fibrinolysis components between patients with only local bleeding and those without bleeding. We also observed that the median levels of factor V and plasminogen were lower in patients with systemic bleeding than in those with only local bleeding. On the other hand, there was no statistically significant difference in the median levels of factor VII and factor X between all groups. Although the median platelet count was within the normal range in *B. atrox* snakebite patients, it was lower in patients with systemic bleeding when compared to those with local bleeding alone and the HD group.

### 2.4. Venom Detection and Hemostatic Parameters 

In 35/100 patients, serum levels of *Bothrops* venom above the cut off value (18.87 ng/mL) were detected. There was no statistically significant difference in the serum level of venom between patients with systemic bleeding (median = 21.10 ng/mL serum (IQR, 5.02–52.35)) and those without bleeding (median = 7.50 ng/mL serum (IQR, 2.50–16.40)) (*p* = 0.209). Moreover, there was also no statistically significant difference in the serum level of venom between patients with systemic bleeding and only local bleeding (median = 19.10 ng/mL serum (IQR, 3.75–32.03)) (*p* = 0.675). Serum levels of *Bothrops* venom were significantly higher in those patients with thrombocytopenia (median = 28.20 ng/mL serum (IQR, 21.35–64.25)) than those who did not present this alteration (median = 7.75 ng/mL serum (IQR, 3.10–23.68)) (*p* = 0.021). Furthermore, there was no significant difference in the serum level of venom between mild cases (median = 22.35 ng/mL serum (IQR, 5.95–30.60)) and moderate/severe cases (median = 7.70 ng/mL serum (IQR, 3.30–22.40)) (*p* = 0.339).

## 3. Discussion

Bleeding and alterations in coagulation are the most common hemostatic disorders and frequently coexist in victims of *Bothrops* snakebite [16,27]. These disorders are caused by the action of venom’s hemostatically active toxins, wherein the variability of these toxins can lead to different mechanisms of coagulopathy [22] and bleeding. While trying to understand how clotting factors and other components are affected in *B. atrox* snakebite patients who evolve to systemic or local bleeding, we found that patients with systemic bleeding presented lower levels of factor V, II, fibrinogen, plasminogen and alpha 2-antiplasmin, and higher levels of tissue factor and FDP than those without bleeding. On the other hand, patients with only local bleeding showed coagulation factor levels and fibrinolysis components similar to those without bleeding. 

*B. atrox* is responsible for 80–90% of snakebites in the Brazilian Amazon [18,28]. Despite the similarities between the toxins from the *B. atrox* and *B. jararaca* venoms [6,26], in our study, we observed that 20.0% (20/100) of *B. atrox* snakebite patients in the western Brazilian Amazon present systemic bleeding, while 46.1% of *B. jararaca* snakebite patients present this manifestation [27]. In a recent study performed by our research group in the western Brazilian Amazon it was observed that unclottable blood and thrombocytopenia were independently associated with the risk of *Bothrops* snakebite patients developing systemic bleeding [16]. In our study, unclottable bood was observed in 95.0% (19/20) of patients with systemic bleeding. Unclottable blood observed in *B. atrox* envenomation can be explained by the presence of components with thrombin-like activity, which directly convert fibrinogen into fibrin [4,5,8] and pro-coagulant toxins, which in turn activate factors II, X and V [10,11,12], and increase the pro-coagulant activity of factor VIII [14], which, as a result, leads to intravascular thrombin generation. The intravascular thrombin generation can also result in activation and consumption of factors V, VIII, XI, XIII, and fibrinogen, as well as thrombocytopenia [29,30]. Furthermore, fibrinogenolytic components of *B. atrox* venom can also contribute to fibrinogen consumption [15]. 

In our study, one interesting and novel result obtained was the detection of increased levels of plasma tissue factor in *B. atrox* snakebite patients who presented systemic bleeding. Although this has never been previously described for *Bothrops* snakebite patients, this result corroborates previous findings in animal and in vitro models, in which *Bothrops* venom and its procoagulant toxins induce tissue factor expression [31,32,33]. Thus, tissue factor could also contribute to the development of unclottable blood and thrombocytopenia, by leading to the intravascular generation of thrombin, and systemic bleeding. In our study, plasma tissue factor presented weak correlation to factor V, factor II, D-dimer, plasminogen, and alpha 2-antiplasmin, and moderate correlation to fibrinogen and FDP. Moreover, the median levels of plasma tissue factor were increased in patients with moderate/severe edema. Tissue factor is a transmembrane protein expressed constitutively by subendothelial cells (extravascular compartment) or induced by monocytes and endothelial cells (intravascular compartment), and it is present in plasma through the release of soluble tissue factor or in microvesicles [34]. It acts as a cofactor for factor VII, whose complex activates factors X and IX, and triggers blood coagulation [35]. Our finding shows a possible role of *Bothrops* venom in inducing the expression of intravascular tissue factor, as well as extravascular tissue factor released by local trauma and damage caused by envenomation. Considering that activators of factors X and II were found in the *B. atrox* venom [10,11,22] and the tissue factor expression detected in our patients, our results indicate that these factors are triggered during *B. atrox* envenomation. However, the normal median values of factors X and VII in our patients suggest that only minute amounts are required to induce coagulopathy, or it may be that an increased hepatic synthesis of these factors may occur in stressful situations [36]. Additionally, high levels of factor VII have been reported in envenomation caused by other snakes [37].

Our results showed that patients with systemic bleeding presented increased levels of FDP, as well as decreased levels of plasminogen and alpha 2-antiplasmin, when compared to those without bleeding. *B. atrox* venom could be acting directly on fibrin and fibrinogen resulting in the generation of fibrinolysis products, and indirectly inducing the release of tissue plasminogen activator [6,15,38,39]. Also, the capacity of *B. atrox* venom to induce factor XIII activation [14], and intravascular thrombin generation [30] can contribute to the formation of intravascular cross-linked fibrin polymer and D-dimer. Similarly, the decreased levels of alpha 2-antiplasmin indicate its consumption in order to inhibit generated plasmin [40]. Consequently, the response of fibrinolysis is essential to eliminate fibrin deposition, thus avoiding microvascular thrombotic obstruction, and resulting end-organ damage or organ failure [41].

Our findings demonstrated that platelet count presented a weak correlation to plasma tissue factor levels and factor II and plasminogen activity in *B. atrox* snakebite patients. Activated platelets are found to enhance tissue factor expression in monocytes, and also expose intracellular stored tissue factor on their own surface [42]. *B. jararaca* envenomation in rats has been shown to trigger the coagulation cascade, which induces direct activation of coagulation factors and tissue factor decryption, and both factors probably contribute to the consumptive coagulopathy [43]. Moreover, platelets also serve as assembly sites for proteins of the prothrombinase complex and plasminogen activator system [44,45], which explains the correlations observed, although they are weak. On the other hand, our results did not show a correlation between fibrinogen levels and platelet count either, similar to what is observed in *B. jararaca* envenomation [27]. Although the median platelet count was within the normal range, it was significantly decreased in patients with systemic bleeding when compared to patients with only local bleeding and healthy donors. Thrombocytopenia was observed in about 68% of *B. jararaca* snakebite patients with systemic bleeding [20,27]. In contrast, only 25% of patients with systemic bleeding were thrombocytopenic in our study. *B. atrox* venom is composed of proteins, such as botrocetin [9], and thrombocytin (a serine proteinase) [14], which induce platelet agglutination or aggregation, respectively, as well as components with platelet inhibiting properties, such as batroxrhagin (a metalloproteinase) [6] and batroxostatin (an Arg-Gly-Asp-containing peptide) [13]. In addition, crude *B. atrox* venom has no aggregating activity on washed rabbit platelets [46]. Moreover, the major toxic component in the venom of *B. atrox* from the Brazilian Amazon, called batroxrhagin, inhibits collagen-induced platelet-aggregation, induces bleeding and fibrin lysis [6]. In *B. jararaca* snakebite patients, disturbances of platelet function also contribute to the development of bleeding due to the envenomation [47]. Changes in platelet function were not evaluated in our study. Therefore, the systemic bleeding observed in *B. atrox* snakebites patients from the Brazilian Amazon may be associated with the impairment of platelet function.

In our study, the levels of venom in the serum of patients were higher in patients with thrombocytopenia than those with normal platelet count. Thus, this finding could be the result of the intravascular thrombin generation induced by the coagulant action of the venom [30], since the main toxin in *B. atrox* venom is an inhibitor of platelet aggregation [6]. On the other hand, there was no association between venom levels and systemic bleeding, or between venom levels and clinical severity of envenomation. Venom has been detected in blister aspirates collected from the bite site in *Bothrops* snakebite patients [18,48]. Moreover, local manifestations such as severe edema, blister formation, and necrosis at the bite site have been observed in *Bothrops* envenomations, which were classified as moderate/severe cases [49]. The most abundant components found in venoms from the *B. atrox* of Brazilian Amazon are metalloproteinases, c-type lectin-like, phospholipases A_2_ and serine proteinases, and are frequently involved in the hemostatic disorders observed in snakebite patients [26,50,51]. Among them, venom hemorraghins, which degrade capillary basement membrane components and induce endothelial disrupture, and have an important role in the development of the bleeding disorders since increased serum levels of this toxin have been associated with systemic and/or local bleeding in *B. jararaca* snakebites patients [52,53]. Thus, the systemic and/or local bleeding observed in our study could also be associated with increased serum levels of hemorraghin. However, serum hemorraghin levels were not measured in our study. 

In conclusion, the systemic bleeding observed in *B. atrox* envenomations in this part of the Brazilian Amazon is a multifactorial outcome. A complex pathophysiological mechanism involving thrombocytopenia, unclottable blood, and endothelial disrupture contributes to the development of systemic bleeding. A new and surprising finding was that plasma tissue factor is involved in the pathophysiology of *B. atrox* envenomation with systemic bleeding in the Amazon region, which shows that the extrinsic coagulation pathway also contributes to the coagulopathy in this type of envenomation.

## 4. Materials and Methods 

### 4.1. Study Design

This was an observational study designed to evaluate the changes in coagulation factor levels, fibrinolysis components, and platelet count associated with bleeding in *B. atrox* snakebite patients in the western Brazilian Amazon. This study was conducted in accordance with the Declaration of Helsinki, and the study protocol was approved by the Ethics Review Committee at the Dr Heitor Vieira Dourado Tropical Medicine Foundation, Manaus, Amazonas State, Brazil (approval number: 1.302.174, approval date: 29 October 2015). All participants and their parents/guardians (for minors) provided written and informed consent.

### 4.2. Participants and Data Collection

We evaluated 100 patients who had received a clinical–epidemiological diagnosis of *Bothrops* envenomation and who were treated at the Dr Heitor Vieira Dourado Tropical Medicine Foundation, between January 2016 and December 2017. These patients were also included in another study performed during this same period [19]. Victims that had undergone previous antivenom therapy at other health service centers or were ≤12 years old age were not included in the study. Of the one hundred patients, thirty-six patients brought the snake that caused the snakebite to hospital. All thirty-six snakes were identified as *B. atrox*. In nineteen patients who did not bring the snake to the hospital, *Bothrops* envenomation was confirmed by venom-specific enzyme immune assays (EIA). In forty-five patients, confirmation of *Bothrops* envenomation was based only on the clinical–epidemiological diagnosis.

In addition, 14 healthy donors of either gender, with no history of snakebite and who were accompanying the patients during hospitalization, comprised the control group. For this study, patients had additional serum and citrate plasma samples taken, and these were collected on admission (before antivenom therapy). All serum and plasma samples were immediately centrifuged after the collection, then frozen and stored at −80 °C.

A standard form was filled in with patient characteristics such as gender, age, time taken to reach medical assistance, anatomical location of the bite, pre-hospital treatment (use of topical or oral medicines, use of a tourniquet and other procedures), *Bothrops* snakebite confirmation by snake identification, presence of systemic and/or local bleeding (before or after of the antivenom therapy), presence of comorbidities, edema (absent, mild (affecting 1–2 limb segments), moderate (affecting 3–4 limb segments) and severe (affecting 5 limb segments) [54]) and clinical severity of envenomation (mild, moderate, or severe cases according to the Brazilian Ministry of Health guidelines) [55,56]. 

### 4.3. Laboratory Analysis

Clotting time was determined by the 20 min whole blood clotting test [57,58,59]. Assays of coagulation factors and fibrinolysis components were carried out on plasma samples obtained from venous blood containing sodium citrate as the anticoagulant and 2% v/v *Bothrops-Lachesis* antivenom (to neutralize *Bothrops* venom present in the sample). The plasma tissue factor was measured by the human EIA kit (Abcam, Cambridge, UK). Plasma factor VII was estimated using deficient plasma kits (Diagnostica Stago S.A.S., Asnières sur Seine, France) with a semi-automatic Start4 coagulation analyzer (Diagnostica Stago S.A.S., Asnières sur Seine, France). Levels of plasma factors II and X were determined by performing a modified prothrombin time test using deficient plasmas kits (HemosIL^®^, Instrumentation Laboratory, Bedford, MA, USA) with an ACL TOP 300 CTS coagulation analyzer (Werfen Instrumentation Laboratory, Barcelona, Spain). Plasma factor V was determined using the Denson standardized method [60]. Plasma fibrinogen was measured based on the Clauss method [61], using a fibrinogen-C kit (HemosIL^®^, Instrumentation Laboratory, Bedford, MA, USA), with an ACL TOP 300 CTS coagulation analyzer (Werfen Instrumentation Laboratory, Barcelona, Spain). Plasma FDP was qualitative and semi-quantitative, and determined by latex agglutination (Diagnostica Stago S.A.S., Asnières sur Seine, France). Plasma D-dimer was measured by immunoturbidimetry, using a D-Dimer HS 500 kit (HemosIL^®^, Instrumentation Laboratory, Bedford, MA, USA), with an ACL TOP 300 CTS coagulation analyzer (Werfen Instrumentation Laboratory, Barcelona, Spain). Plasma plasminogen was determined by chromogenic assay (American Diagnostica Inc., Greenwich, UK). Alpha 2-antiplasmin was determined by the synthetic chromogenic substrate method (Diagnostica Stago S.A.S., Asnières sur Seine, France). These assays were performed according to the manufacturer’s protocols at the Hemostasis Laboratory, Amazonas State Hematology and Haemotherapy Hospital Foundation (dosage of factors II and X, fibrinogen and D-dimer) and Pathophysiology Laboratory at the Butantan Institute (dosage of tissue factor, factors V and VII, plasminogen, FDP, and alpha 2-antiplasmin). Platelet counts were carried out on venous blood samples containing potassium EDTA as anticoagulant and 2% v/v *Bothrops-Lachesis* antivenom, and determined in an automated cell counter (Sysmex Corp., Kobe, Japan), at the Carlos Borborema Clinical Research Institute, Dr Heitor Vieira Dourado Tropical Medicine Foundation.

EIA was used for the detection and measurement of *Bothrops* venom circulating in serum samples. Antigens were captured with *Bothrops-Lachesis* antivenoms coated on ELISA plates, and the bound toxins reacted with a second antibody raised in rabbits against *B. atrox* venom, followed by incubation with anti-rabbit IgG-peroxidase conjugate. Reactions were revealed by the addition of the enzyme substrates (OPD plus H_2_O_2_) and recorded at 492 nm. Values were calculated using standard curves with known concentrations of *B. atrox* venom. The cut-off level corresponded to the mean plus 2 S.D. values of negative samples obtained from the HD group. This assay was performed at the Immunopathology Laboratory, Butantan Institute.

### 4.4. Outcomes

The laboratory outcomes consisted in the presence of (i) unclottable blood, and (ii) thrombocytopenia on admission (before antivenom therapy). Unclottable blood was defined as when the blood had not clotted within 20 min after collection. Thrombocytopenia was defined by having a platelet count below 150 × 10^3^/µL. The clinical outcome was the presence of systemic and/or local bleeding on admission (before antivenom therapy), or during hospitalization (after antivenom therapy). Local bleeding was determined as the presence of local ecchymosis; bleeding from the fang punctures.

### 4.5. Data Analysis

A database and descriptive analyses were produced using Microsoft Excel Office 365 software, which was fed information by two independent typists. Statistical analyses were performed using the Graphpad Prism software version 5 (Graphpad Software, Inc., San Diego, CA, USA) and STATA statistical package version 13 (Stata Corp, College Station, TX, USA). The comparison of medians between two data groups were performed using the Mann–Whitney test, whereas for comparison of the variables with three or more groups, the data analysis was performed using the Kruskal–Wallis test, followed by Dunn’s post-test for multiple comparisons between groups. Differences in results were considered statistically significant when *p* < 0.05. Data normality tests were done using the Shapiro–Wilk test. Pearson’s correlation coefficient was calculated to measure the degree of association between the platelet count and coagulation factor levels and fibrinolysis components. Spearman’s correlation coefficient was calculated to measure the degree of association between the tissue factor and coagulation factor levels/fibrinolysis components and serum venom. The coefficient correlation factor was considered significant when *p* < 0.05. The correlation index (*r*) was used to categorize the correlation strength as being weak (*r* ≤ 0.35), moderate (*r* ≥ 0.36 to *r* ≤ 0.67), or strong (*r* ≥ 0.90), as previously described [49,62].

## Figures and Tables

**Table 1:** Background information and clinical features of the *Bothrops atrox* snakebite patients (*n* = 100) at the time of admission, based on the presence of bleeding.

	Systemic Bleeding	Only Local Bleeding	Non-bleeding
**Characteristics**	***n* (%)**	***n* (%)**	***n* (%)**
**Gender**			
Male:female	18:2	35:6	35:4
**Age (median) years (range)**	33 (15–58)	42 (14–74)	48 (15–79)
**Time taken to reach to medical assistance (hours)**			
<3 (*n* = 46)	7 (15.2)	20 (43.5)	19 (41.3)
3–6 (*n* = 34)	5 (14.7)	17 (50.0)	12 (35.3)
>6 (*n* = 20)	8 (40.0)	4 (20.0)	8 (40.0)
**Anatomical location of bite**			
Foot (*n* = 73)	16 (21.9)	30 (41.1)	27 (37.0)
Leg (*n* = 18)	2 (11.1)	6 (33.3)	10 (55.6)
Hand (*n* = 9)	2 (25.0)	5 (50.0)	2 (25.0)
**Edema**			
Mild (*n* = 46)	8 (17.4)	16 (34.8)	22 (47.8)
Moderate (*n* = 49)	8 (16.3)	25 (51.0)	16 (32.7)
Severe (*n* = 5)	4 (80.0)	0 (0.0)	1 (20.0)
**Pre-hospital treatment**			
Tourniquets (*n* = 24)	5 (20.8)	12 (50.0)	7 (29.2)
Local incisions (*n* = 4)	1 (25.0)	2 (50.0)	1 (25.0)
Suction (*n* = 1)	0 (0.0)	1 (100.0)	0 (0.0)
Use of topical/oral medicines (*n* = 55)	14 (25.5)	22 (40.0)	19 (34.5)
**Comorbidities**			
Arterial hypertension (*n* = 16)	1 (6.3)	8 (50.0)	7 (43.7)
Diabetes (*n* = 6)	1 (25.5)	1 (25.5)	4 (50.0)
Cardiopathy (*n* = 1)	0 (0.0)	1 (100.0)	0 (0.0)
**Clinical severity of envenomation**			
Mild (*n* = 27)	1 (3.7)	11 (40.7)	15 (55.6)
Moderate (*n* = 59)	14 (23.7)	22 (37.3)	23 (39.0)
Severe (*n* = 14)	5 (35.7)	8 (57.1)	1 (7.1)
**Unclottable blood**			
Yes (*n* = 54)	19 (35.2)	21 (38.8)	14 (25.9)
No (*n* = 46)	1 (2.2)	20 (43.5)	25 (54.3)
**Thrombocytopenia**			
Yes (*n* = 10)	5 (50.0)	2 (20.0)	3 (30.0)
No (*n* = 90)	15 (16.7)	39 (43.3)	36 (40.0)
